# Heterogeneity in SDF-1 Expression Defines the Vasculogenic Potential of Adult Cardiac Progenitor Cells

**DOI:** 10.1371/journal.pone.0024013

**Published:** 2011-08-24

**Authors:** Claudia O. Rodrigues, Lina A. Shehadeh, Michael Hoosien, Valerie Otero, Ines Chopra, Nicholas F. Tsinoremas, Nanette H. Bishopric

**Affiliations:** 1 Department of Molecular and Cellular Pharmacology, University of Miami Leonard M. Miller School of Medicine, Miami, Florida, United States of America; 2 Department of Medicine, Division of Cardiology, University of Miami Leonard M. Miller School of Medicine, Miami, Florida, United States of America; 3 Center for Computational Sciences, University of Miami Leonard M. Miller School of Medicine, Miami, Florida, United States of America; Brigham and Women's Hospital, United States of America

## Abstract

**Rationale:**

The adult myocardium has been reported to harbor several classes of multipotent progenitor cells (CPCs) with tri-lineage differentiation potential. It is not clear whether c-kit+CPCs represent a uniform precursor population or a more complex mixture of cell types.

**Objective:**

To characterize and understand vasculogenic heterogeneity within c-kit+presumptive cardiac progenitor cell populations.

**Methods and Results:**

c-kit+, sca-1+ CPCs obtained from adult mouse left ventricle expressed stem cell-associated genes, including Oct-4 and Myc, and were self-renewing, pluripotent and clonogenic. Detailed single cell clonal analysis of 17 clones revealed that most (14/17) exhibited trilineage differentiation potential. However, striking morphological differences were observed among clones that were heritable and stable in long-term culture. 3 major groups were identified: round (7/17), flat or spindle-shaped (5/17) and stellate (5/17). Stellate morphology was predictive of vasculogenic differentiation in Matrigel. Genome-wide expression studies and bioinformatic analysis revealed clonally stable, heritable differences in stromal cell-derived factor-1 (SDF-1) expression that correlated strongly with stellate morphology and vasculogenic capacity. Endogenous SDF-1 production contributed directly to vasculogenic differentiation: both shRNA-mediated knockdown of SDF-1 and AMD3100, an antagonist of the SDF-1 receptor CXC chemokine Receptor-4 (CXCR4), reduced tube-forming capacity, while exogenous SDF-1 induced tube formation by 2 non-vasculogenic clones. CPCs producing SDF-1 were able to vascularize Matrigel dermal implants *in vivo*, while CPCs with low SDF-1 production were not.

**Conclusions:**

Clonogenic c-kit+, sca-1+ CPCs are heterogeneous in morphology, gene expression patterns and differentiation potential. Clone-specific levels of SDF-1 expression both predict and promote development of a vasculogenic phenotype via a previously unreported autocrine mechanism.

## Introduction

Heart failure is a lethal and disabling end result of a number of highly prevalent cardiovascular diseases, including hypertension and coronary atherosclerosis, and is estimated to affect 2.8% of the present US population[1]. Although current trends show some improvement in heart failure-specific mortality [2], [3], [4], the predicted 25% increase in heart failure by the year 2030 will pose a major therapeutic challenge. Recent basic research studies have shown that myocyte loss plays a major role in the induction and progression of most if not all forms of heart failure [5], [6], [7], [8], [9], [10]. In parallel, studies have revealed that many adult tissues, notably bone marrow, but also skeletal muscle, synovium and adipose tissue, contain self-renewing, pluripotent cells capable of repairing injured myocardium and/or improving blood flow to the heart ([11], [12], [13], [14], reviewed in [15]). These insights have led to a rapid and intensive pursuit of regenerative strategies to increase the number of functional cardiac myocytes and blood vessels in the damaged and failing myocardium (reviewed in[16], [17], [18], [19], [20], [21]). In the last 5 years, clinical trials have shown myocardial delivery of stem cells from bone marrow and other sources to be safe and effective in improving clinical outcomes, with generally favorable effects on left ventricular function[22], [23], [24], [25], [26], [27]; further large randomized trials are continuing [28], [29], [30]. However, significant controversy remains. Among other issues, there is no agreement on the mechanism of action of stem cell therapy, nor on the optimal method, dose and timing of their delivery; the best source of reparative cells also has yet to be established[31], [32], [33], [34].

The adult myocardium has recently been shown to harbor multipotent progenitor cells that can give rise to both myogenic and vasculogenic lineages, and that have been shown to contribute to myocardial repair [11], [35], [36], [37], [38]. Several different types of cardiac precursor cells (CPCs) have been described, distinguished by method of isolation and/or expression of surface markers, including c-kit, stem cell antigen (sca-1), transporter protein ABC1, and transcription factor islet-1 (Isl1). Each of these has been reported to be self-renewing, to differentiate along three major myocardial lineages (cardiac myocytes, smooth muscle and endothelial cells) [11], [39], [40], [41], [42], [43], [44], [45], [46], and to be capable of reconstituting injured myocardium [11], [13], [39], [42], [47], [48], [49]. Despite the greater accessibility of other progenitor cell types, cardiac-derived stem cells have excited considerable therapeutic interest, because of their greater potential for cardiomyogenic differentiation, engraftment and survival within the myocardium[34], and the potential of endogenous CPCs to respond to exogenous or paracrine mobilization signals. However, the clinical application of CPCs remains limited by substantial uncertainty over how to define, isolate and expand an optimum cell type for transplantation, and more fundamentally by a need to understand the origins and biological properties of various CPC populations [9], [11], [50], [51], [52].

In this study, we performed single cell cloning to study the properties of one type of cardiac progenitor cell: sca-1+, c-kit+CPCs derived from the left ventricles of adult mouse hearts. We show that these cells exhibit a surprising degree of clonally stable heterogeneity in morphology, gene expression and functional properties, and importantly, in the potential for vasculogenic differentiation. We find that a major component of this heterogeneity is clonal variation in endogenous expression of the chemokine SDF-1, which in turn controls the morphology and vasculogenic potential of CPCs. We conclude that SDF-1 may serve both as a biomarker and an effector of CPC therapeutic potential.

## Results

### Isolation of clonogenic cardiac progenitor cells (CPCs)

**Figure 1:**
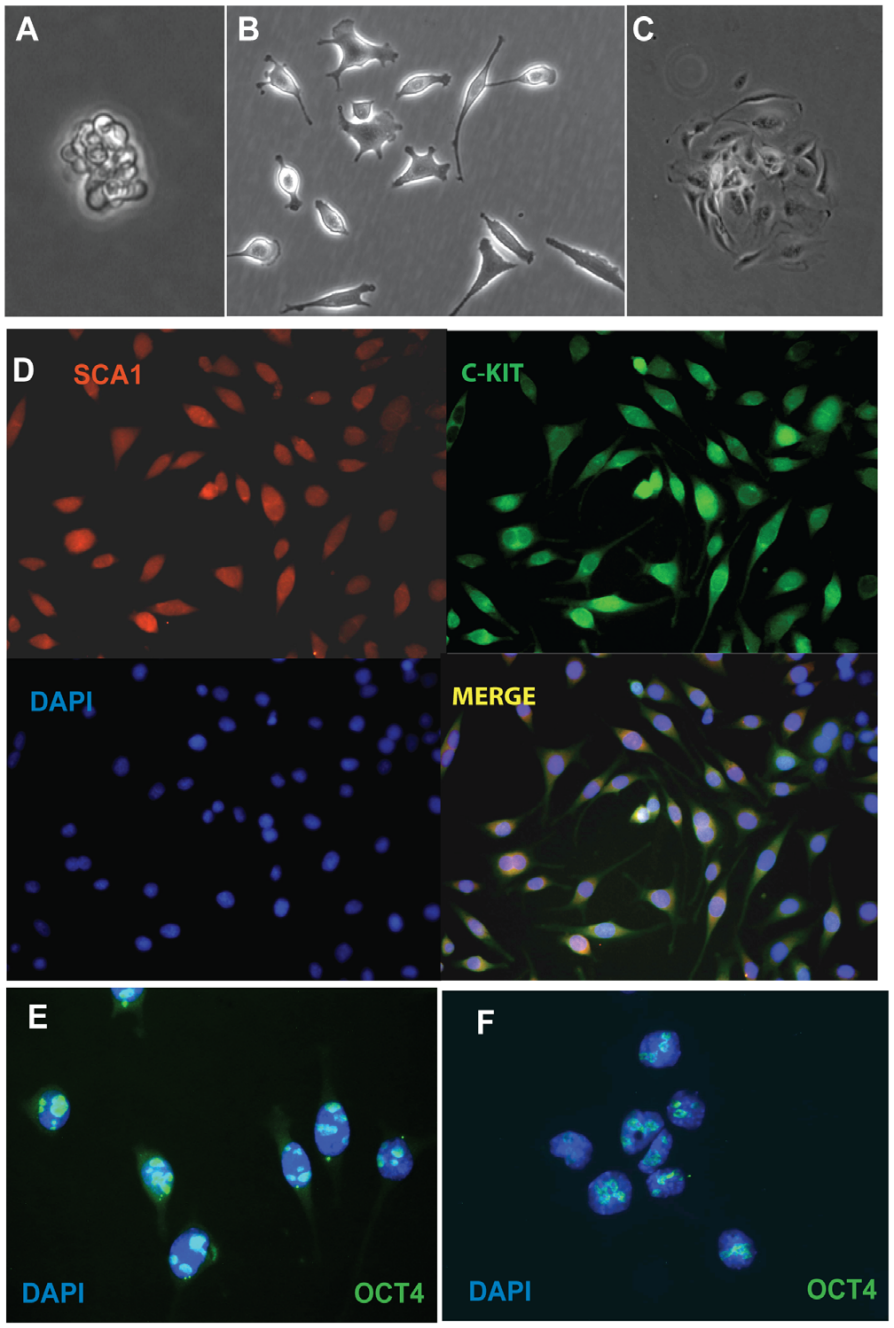
Cardiac progenitor cells express stem cell markers. A. Cardiosphere 24 hours after CPC isolation. B. Morphologically heterogeneous parental CPC isolate. C. CPC colony used for initial clonal expansion. D. CPCs stained for stem cell markers Sca-1 and c-Kit, counterstained with DNA dye DAPI to reveal nuclei. E–F. CPCs (E) and P19 teratocarcinoma cells (F) expressing Oct4 (green). Original magnification A–D: 32x. Original magnification E, F: 100x.

Sca-1+cells were isolated from the left ventricles of adult mice expressing a GFP transgene under the human ubiquitin C (UBC) promoter (C57BL/6-tg(UBC-GFP)30Scha) and cultured in bacterial Petri dishes for 2-days, after which cell aggregates grown in suspension were transferred to tissue culture plates and expanded (Fig. 1A–C). Cells at this stage were positive for both Sca-1 and c-kit (Fig. 1D). Expression of the embryonic stem cell marker Oct4 was readily detected (Figure 1E) and similar to that seen in P19 teratocarcinoma cells (Figure 1F). Serial dilution cloning was used to obtain 65 single cell clones, of which 17 have now been expanded up to 60 times without evidence of senescence, confirming a significant self-renewal capacity. Doubling time was ∼24–48 h for all clones (Figure S1). 17 low passage (P8–P23) clones were selected for further characterization.

### CPCs are morphologically heterogeneous

**Figure 2:**
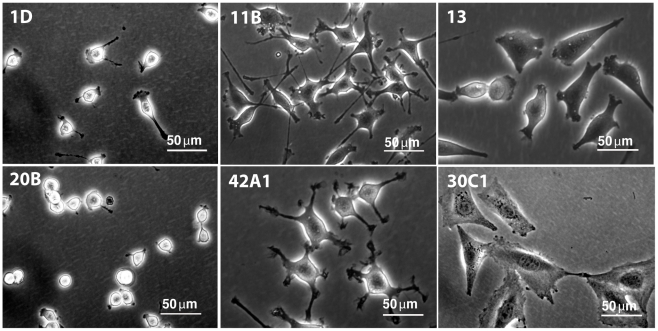
Clonal variation in CPC morphology. Single cell CPC clones from the original isolate showed clonal, stable differences in shape, size, and nucleus/cytosol ratio. Shown are CPC clones representing 3 basic phenotypes: small and round (CL1D and CL20B), stellate (CL11B and CL42A1), and spindle/flat (CL13 and CL30C1).

Cells from the initial isolate exhibited morphological heterogeneity (Figure 1B), falling broadly into 3 categories: spindle and flat, stellate, and small and round, in roughly equal proportions (Figure 2). Stellate cells had multiple cellular projections of varying lengths that occasionally ramified. Small, round clones had few visible cellular projections and a high nucleus:cytosol ratio. Cell volumes in suspension varied little, however (data not shown), suggesting that the observed differences in shape were related to cell-matrix attachments. In all cases, cell morphology was a clonally stable trait that did not change with serial passage.

### CPC clones differ in their commitment to endothelial and muscle lineages

**Figure 3:**
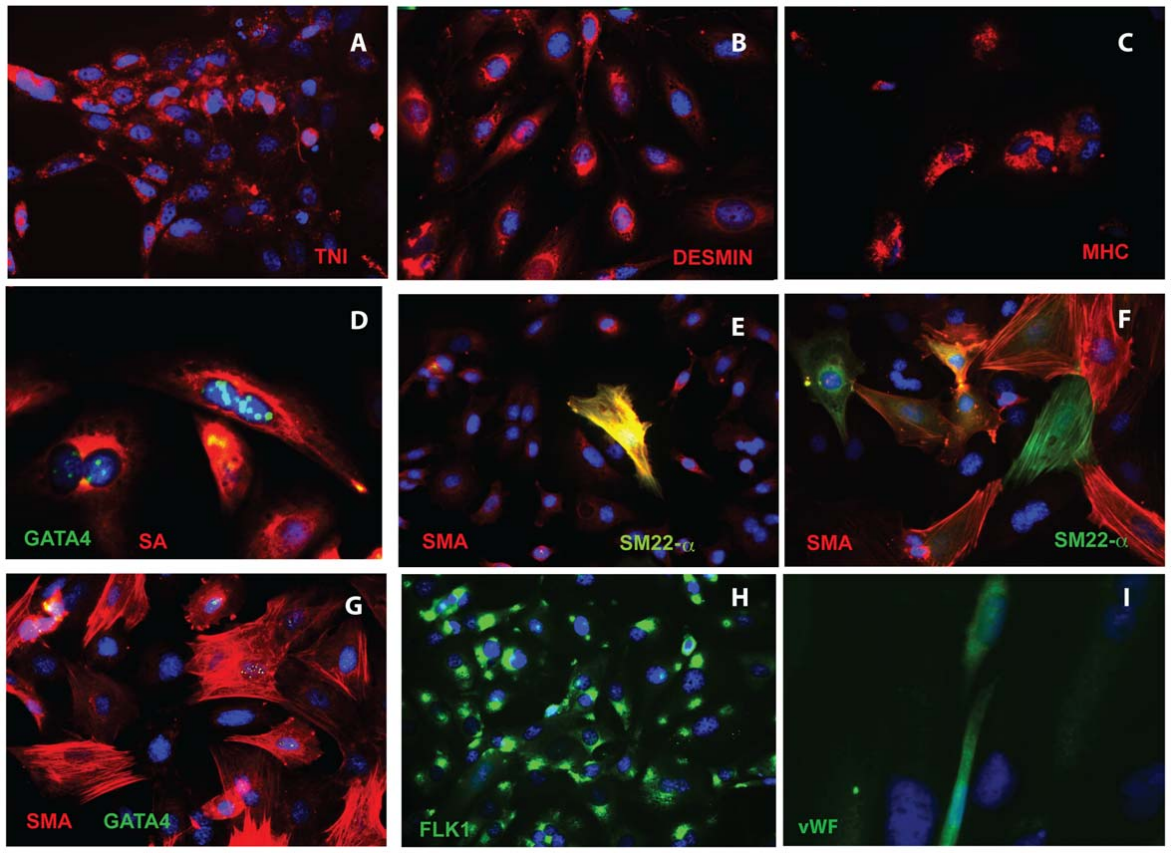
Trilineage differentiation of cardiac progenitor cells. CPCs 4 weeks after differentiation stained with antibodies against cardiac, smooth muscle and endothelial markers as shown. A. TNI. B. Desmin. C. MHC. D. sarcomeric actin (SA) and GATA4. E, F. SMA and SM22-α. G. SMA and GATA4. H. Flk-1/KDR. I. vWF. For images A–C, E-H: original magnification 32X. Images D, I: original magnification 100X. Shown are clones 1D (A, D), 20B (E) and 11B (B, C, F–H).

After 2–4 weeks of differentiation in low mitogen medium, all CPC clones exhibited mesodermal pluripotency, and differentiated in varying proportions along smooth muscle, cardiomyogenic and/or endothelial lineages (Figure 3). 2 clones differentiated along endothelial and cardiac lineages only, and 1 expressed endothelial and smooth muscle but not cardiac markers (see Table 1); the remainder displayed trilineage potential. Cardiac muscle proteins troponin I, desmin, myosin heavy chain and sarcomeric α-actin were induced in some cells (Figure 3A–D), although well-organized sarcomeres and contractile activity were not observed. Some clones gave rise to greater numbers of large, flat cells expressing smooth muscle actin (SMA) organized into filaments (Figure 3F, G), relative to others (Figure 3E). In some of these large cells, SMA co-localized with the smooth muscle marker SM22-α, while in others, expression appeared to be mutually exclusive (compare Figures 3E, F). Most sarcomeric α-actin+ and some SMA+ cells co-expressed GATA4 (Figure 3D, G). All clones contained FLK1+ cells (Figure 3H), both before and after differentiation, and a smaller number of cells expressed von Willebrand Factor (vWF, Figure 3I), supportive of endothelial differentiation.

**Table 1:** Summary of morphological, self-renewal and vasculogenic properties of cardiac progenitor cell clones.

Clone	Morphology	Last Passage #	Lineage differentiation			Vasculogenic index	SDF-1 Levels pg/ml
			Cardiac	Smooth muscle	Endo-thelial		
**CL1D**	small/round	48	−	+	+	17.80	19.09
**CL3**	flat/spindle	23	+	+	+	9.11	12.85
**CL6**	small/round	10	+	+	+	ND	ND
**CL7**	flat/spindle	49	+	−	+	ND	ND
**CL11B**	stellate	60	+	+	+	23.96	157.82
**CL13**	flat/spindle	38	+	−	+	14.50	59.40
**CL17**	stellate	15	+	+	+	21.92	131.57
**CL19**	small/round	17	+	+	+	ND	ND
**CL20**	small/round	15	+	+	+	5.90	29.96
**CL20B**	small/round	50	+	+	+	7.91	37.50
**CL22**	stellate	15	+	+	+	19.40	202.61
**CL23**	small/round	15	+	+	+	18.19	54.12
**CL25**	stellate	11	+	+	+	ND	ND
**CL27**	flat/spindle	14	+	+	+	ND	ND
**CL30C1**	flat	54	+	+	+	1.97	15.82
**CL32**	small/round	15	+	+	+	16.80	45.06
**CL42A1**	stellate	41	+	+	+	9.82	84.91

Morphology was assessed by light microscopy and immunocytology. Last passage number: maximum number of passages to date. Vasculogenic index: length x number of tubes formed per high power field in Matrigel at 5 hours. SDF-1 levels were determined by ELISA (see Methods).

### Morphology, but not FLK-1 expression, identifies CPC subpopulations with enhanced vasculogenic potential

**Figure 4:**
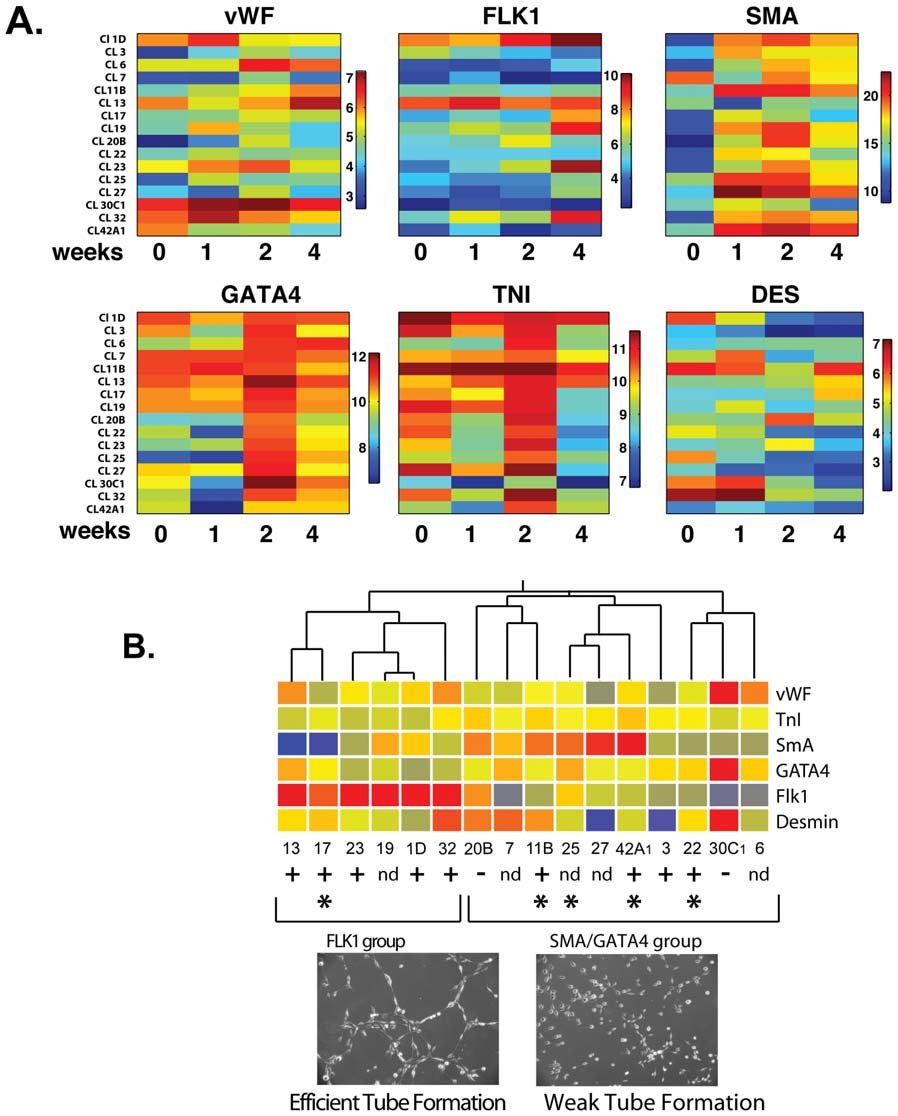
Heterogeneous differentiation of CPC clones. A. Heat map showing clonal heterogeneity in timing and level of induction of lineage markers during differentiation. Absolute values of each mRNA expressed in log2 scale. B. Peak marker gene expression levels were used for unsupervised hierarchical clustering. * stellate, + efficient and (−) weak tube formation. Below: Clones 42A1 (efficient) and 20B (weak) after 5 h in Matrigel.

To further characterize the differentiation potential of individual clones, induction of lineage marker gene expression was followed over a 4-week period after LIF withdrawal in 16 clones. Consistent clone-specific variations were observed in the timing and quantity of induced lineage marker mRNAs (Figure 4A). When lineage marker expression patterns were analyzed using an unbiased hierarchical clustering algorithm, clones were grouped into 2 main clusters, one of which showed greater induction of FLK-1, and the other greater induction of SMA and to a lesser extent GATA4 (Figure 4B). 4 out of 5 stellate clones fell within the second cluster (Figure 4B, *). Surprisingly, FLK-1 expression was not required for functional vasculogenic competence. Multiple clones from both expression groups underwent efficient endothelial differentiation and capillary tube formation in a 3D Matrigel assay (Figure 4B). Moreover, FLK-1 expression was not sufficient for effective vasculogenesis, as some FLK-1-inducing clones were only weakly vasculogenic (Figure 4B, below right). Clones with stellate morphology tended to be strongly vasculogenic; most formed tubes at a rate similar to that of human umbilis (Figure 4B, below left), and more rapidly than the other clone types (28.2±2.5 vs. 17.5±4.0 tubes/field/5 h, p = 0.04).

### SDF-1 expression levels predict CSC morphology

**Figure 5:**
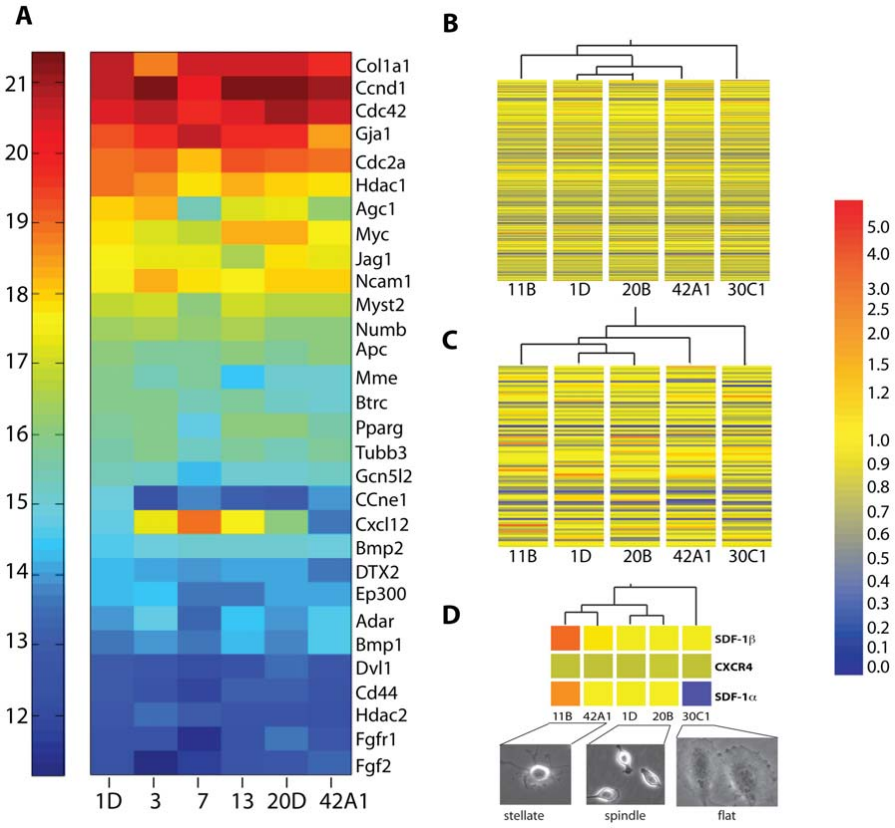
Gene expression associations with morphology. A. Heat map of absolute mRNA expression of stem cell related genes in 6 representative CPC clones. B and C. Clustering by expression levels of (B) all 45,000 genes from array and (C) subset of 84 stem cell-associated genes. D. Clustering by expression of genes in Gene Ontology (GO) “germ cell migration”. Below: Representative images of stellate, spindle and flat morphologies.

In searching for other features that could identify clones with enhanced vasculogenic differentiation, we performed gene expression profiling on a group of 5 undifferentiated clones representing each morphological group (Figure 5). As expected, a focused real-time PCR array of 84 stem cell-related genes showed that 30 were expressed at high levels in each of 6 clones examined, although some heterogeneity was noted, particularly in expression of SDF-1 (Figure 5A). We then performed Affymetrix microarray expression profiling of 3 of these clones plus an additional 2, which confirmed overall similarity in gene expression profiles, with relatively few transcripts showing significant differential expression among clones (Figure 5B; data deposited in GEO database, record # GSE24828). When clones were grouped by gene expression patterns using the same unbiased hierarchical algorithm, the 2 spindle-shaped clones 1D and 20B clustered together, and the single flat clone 30C1 segregated from the other 4, whether all 39,000 genes from the Affymetrix array (Figure 5B) or the subset of stem cell genes (Figure 5C) was used. These results supported an association between gene expression and cell morphology.

To refine the search, we conducted additional hierarchical clustering on subsets of genes defined by biological process terms from Gene Ontology (GO) (http://www.geneontology.org/). 14 GO biological processes, including cell adhesion, microtubule stabilization, and nitric oxide-mediated signal transduction correctly grouped the stellate, spindle and flat clones (Table S1). In particular, stellate morphology was closely associated with elevated expression of SDF-1α and -β isoforms (GO term “germ cell migration”; Figure 5D and Table S1).

### Autocrine SDF-1 production determines CPC vasculogenic properties

**Figure 6:**
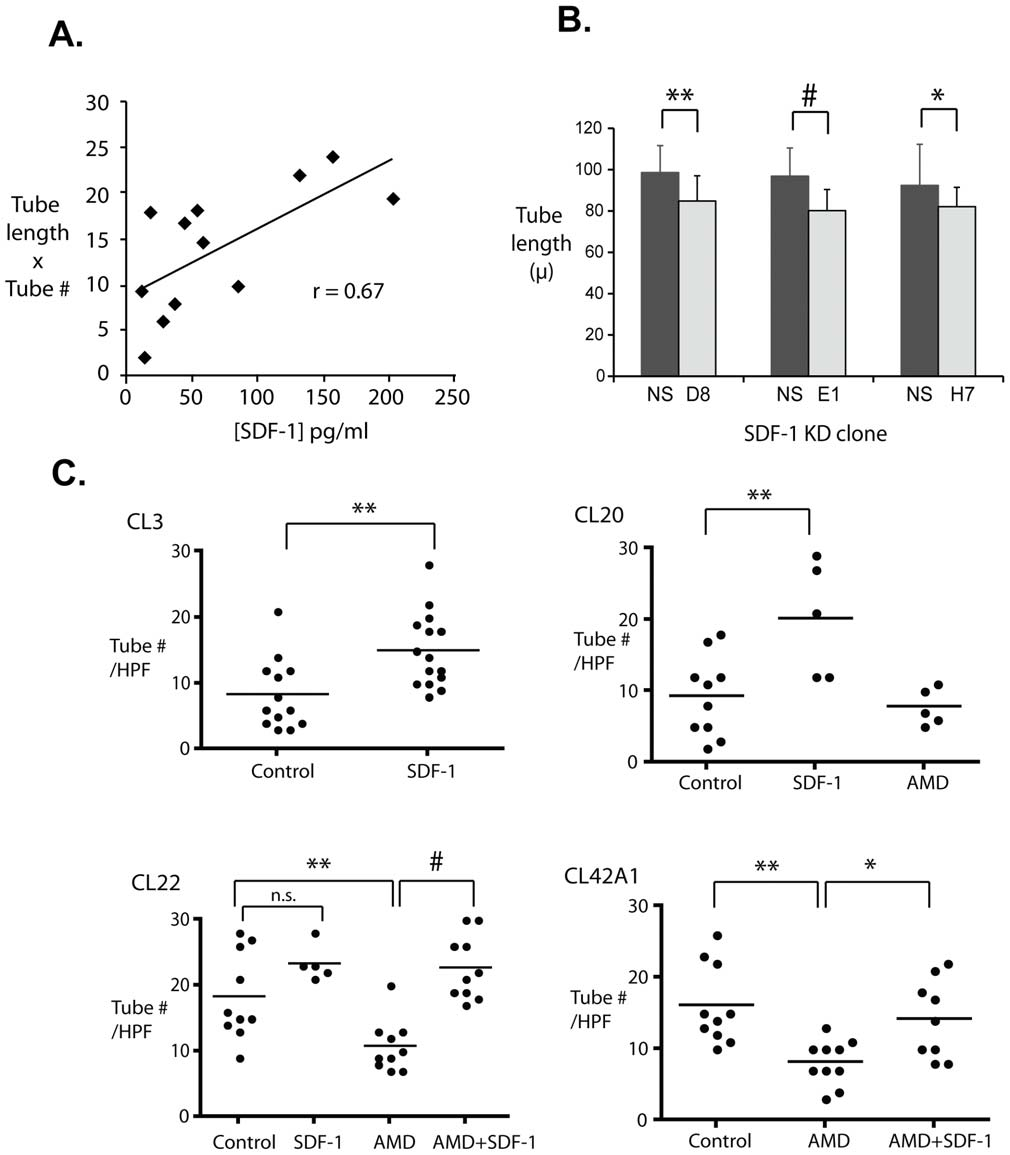
Autocrine SDF-1 signaling determines vasculogenicity of CPC clones in vitro. A. Correlation between SDF-1 expression and vasculogenic potential in Matrigel. B. SDF-1 knockdown reduces CPC tube length in Matrigel. Clones were stably transfected with one of three different SDF-1 shRNAs (D8, E1 and H7) or a scrambled control (NS). C. Exogenous SDF-1 increased mean tube number in both weak (CL3 and CL20) and strong (CL22 and 42A1) tube forming clones, and AMD3100 decreased tube number in strong tube formers (CL22, CL42A1). Dots represent single field counts; n = 5–15 field counts per clone. *p<0.05, **p<0.01, #p<0.001; n.s.  =  not significant.

We reasoned that SDF-1 could be a common driver both of the stellate morphology and of enhanced tube-forming capacity in Matrigel. To determine the relationships among SDF-1, morphology and vasculogenic potential, we measured pre-differentiation SDF-1 and Flk-1 protein in the 12 previously analyzed clones (see Figure 4) and correlated them with quantitative measures of tube formation in Matrigel. SDF-1 protein levels correlated well with tube length and number (r = 0.67) (Figure 6A; see also Table 1). In contrast, FLK-1 protein levels varied widely, from nearly undetectable to high, comparable to HUVECs, but did not correlate with vasculogenic capacity (r = 0.24) (Figure S2).

We next sought to validate the apparent correlation between endogenous SDF-1 production and tube formation, and determine its mechanistic significance. Stable lentivirus-mediated shRNA transfection was used to knock down SDF-1 in one of the highest-expressing clones, CL11B. 45–50% reduction in SDF-1 was achieved by each of 3 different targeting shRNAs (Figure S3). In each case, partial SDF-1 loss resulted in significant reduction in tube length, relative to clones transduced with a non-silencing shRNA (p≤0.05, Figure 6B).

CPC clones were next treated with SDF-1 or AMD3100, an antagonist of the receptor for SDF-1, CXCR4. As predicted, exogenous SDF-1 promoted tube formation in all poorly vasculogenic clones tested, for example CL3 (control *vs.* SDF, 8.38±1.48 *vs.* 15.1±1.46 tubes/HPF, n = 15, p <0.005) and CL20 (9.30±1.8 *vs.* 20.3±3.6, n = 5, p<0.01, S.E.M.) (Figure 6C). Correspondingly, treatment with AMD3100 impaired tube formation in strongly vasculogenic clones, including CL22 (control *vs.* AMD, 18.4±2.1 *vs.* 10.8±1.2, n = 10, p<0.01) and CL42A1 (16.1±1.8 *vs.* 8.20±0.10, n = 10, p<0.01) in a manner that was reversed by exogenous SDF-1 (CL22: AMD *vs.* AMD+SDF-1, 10.8±1.2 *vs.* 22.8±1.5, n = 10, p<0.001; CL42A1: 8.20±0.10 *vs.* 14.2±1.8, n = 10, p<0.05) (Figure 6C). Similar results were obtained with CL17 and CL30C1 (not shown).

### CPC SDF-1 promotes vasculogenesis in dermal implants in vivo

**Figure 7:**
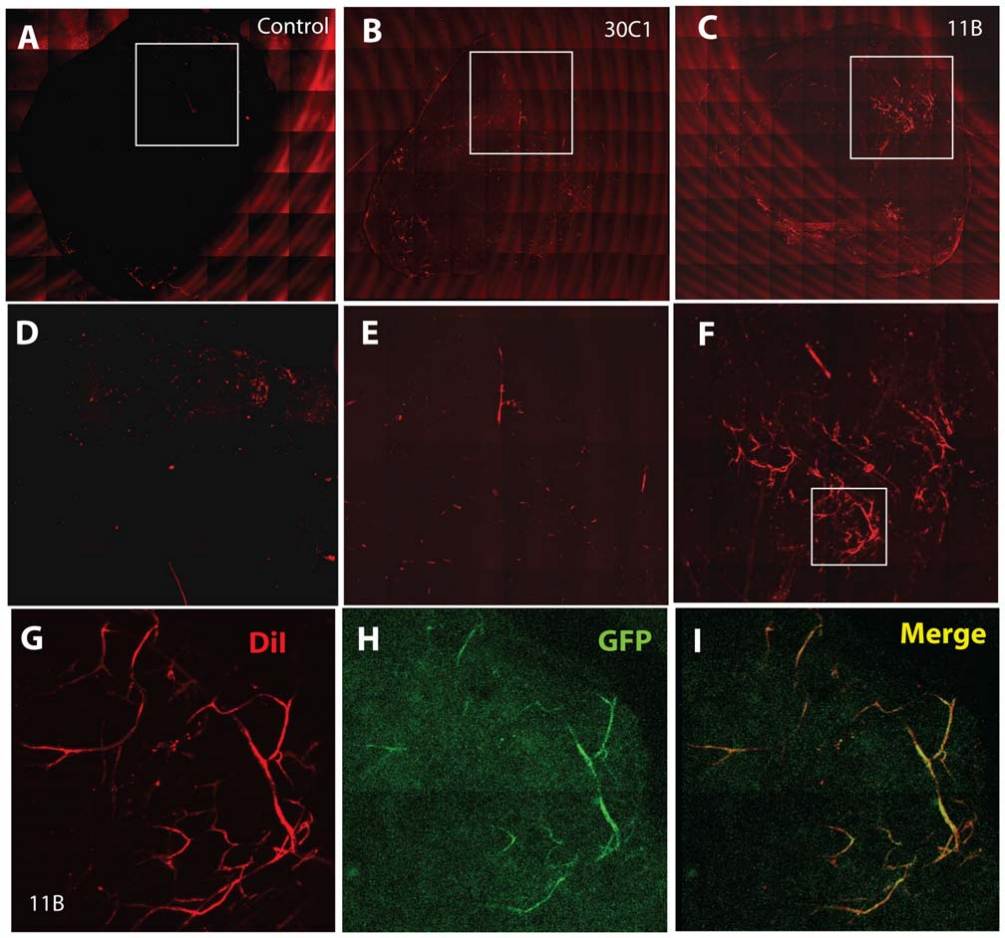
Clonal heterogeneity of CPC vasculogenic properties *in vivo*. A–C. Vascular differentiation of SDF-1-producing CPCs in vivo. Whole mount scan of Matrigel plugs containing no cells (control, A), low SDF-1 clone 30C1 (B) or high SDF-1 clone 11B (C). D–F: Enlargements of boxed areas as shown. G, H: Vascular structures in CPC-seeded Matrigel plugs. Representative fluorescence microscope images of plugs containing clones 30C1 (G) or 11B (H) under identical *in vivo* conditions. I. Overlap of DiI-stained and GFP-expressing blood vessels. Additional images are provided in Figure S4.

To further validate these findings, we performed an *in vivo* assay in which Matrigel inserts were implanted subdermally for one week in congenic C57Bl/6 mice. Matrigel inserts alone (Figure 7A, D), or containing cells from the low SDF-1/non-stellate/weak tube-forming clone 30C1 (Figure 7B, E), were not vascularized. However, inserts with cells from high SDF-1/stellate/strong tube-forming clone 11B reproducibly acquired multiple blood vessels that were continuous with the host circulation (Figure 7C, F-I; Figure S4). Portions of the formed blood vessels within the insert were positive for GFP (Figure 7H, I), indicating a contribution of the GFP transgene-labelled CPCs to these structures. These findings are consistent with our previous findings *in vitro*, and support the view that endogenous SDF-1 production by CPCs enhances their vasculogenic differentiation potential *in vivo,* as well as anastomosis with host-derived blood vessels.

## Discussion

In the adult myocardium, primitive cells capable of regenerating myocytes and, to a lesser extent, resistance coronary arterioles and capillaries in vivo in response to injury have been identified using several different surface markers and isolation techniques[51], [53], [54], [55]. Here we confirm that adult myocardial cells defined by surface expression of Sca-1+ and c-Kit+, described by other groups[11], [42], [45], [49], are pluripotent as defined by induction of distinct lineage markers in individual progeny during in vitro differentiation, and clonogenic, indicating that they represent a true progenitor population. In addition, using single cell clonal analysis, we reveal that these cells comprise multiple subpopulations exhibiting substantial heterogeneity in gene expression profile, morphology and lineage preference, particularly in acquisition of a functional vasculogenic phenotype. Finally, we provide a molecular basis for part of this variability by demonstrating inherent differences in expression of the chemokine SDF-1 that drives morphological and functional angiogenic differentiation. This observation is important because the ability of CPCs to form vascular structures is likely to be key to the support of cell survival and engraftment in the ischemic myocardium, and therefore to their therapeutic usefulness. Our data indicate that SDF-1 expression may be an important way to qualify the angiogenic potential of therapeutic cell isolates for cardiovascular disease.

Vessel wall-resident progenitor cells have been documented in a number of adult tissues, including the bone marrow, skeletal muscle and adipose tissue, and give rise to both endothelial and smooth muscle cells that contribute to post-natal angiogenesis and tissue repair[56]. Recently, a c-kit+ presumptive coronary artery progenitor population was identified by Bearzi et al within the coronary artery wall, that was able to regenerate larger (1.5 mm) resistance vessels and contribute to improved myocardial blood flow in a dog model of ischemia[52]. The cells described here are distinct from the latter, based both on site of isolation (muscle *vs.* blood vessel) and surface expression of Flk-1/KDR, a defining feature of the coronary stem cell that was highly variable in our cell populations.

Phenotypic heterogeneity has been previously noted in primary isolates of mesenchymal stem cells from bone marrow and synovium as well as in myocardial progenitor cells [11], [57], [58]. It is not clear whether this diversity indicates the presence of multiple unrelated cell populations or different stages of differentiation in a single primitive cell type. Our single cell clonal analysis provides a number of important insights into this question. First, we find that the differences in shape are clonally stable and likely dictated by differences in substrate attachment and spreading properties, as cell volumes are essentially identical. Second, although gene expression patterns were generally highly similar, clonally stable differences in expression of specific genes could be demonstrated, possibly reflecting changes acquired by the progeny of a single parental cell type. Cell morphology can be decisively influenced by differences in expression of a few genes, for example, those involved in cytoskeletal organization [59]. Thus, it is plausible that minor clonal changes in the epigenome of progenitors, perhaps linked to local tissue signals, could lead to substantial phenotypic heterogeneity.

SDF-1 (also known as CXCL12) is a chemokine that plays an important role in immune cell attraction, stem cell homing and cancer metastasis [60], [61], [62], [63], [64]. Importantly, SDF-1 also has a direct role in angiogenesis and vasculogenesis[65] and is constitutively expressed by endothelial cells as well as stromal cells from a number of tissues, neural cells and osteoblasts[60], [66]. Loss of SDF-1 or its receptors CXCR4 and CXCR7 leads to defects in vascular development and formation [67], [68], [69]. SDF-1 promotes vascular morphogenesis and sprouting of endothelial cells[66], [70], [71], [72] as well as vascular sprouting from embryoid bodies and aortic rings [73], [74]; SDF-1-CXCR4 signalling plays a critical role in tumor angiogenesis in vivo[75], [76]. SDF-1 promotes de novo vasculogenesis by enhancing the survival, migration, engraftment and differentiation of endothelial precursor cells [77], [78], [79], and supports therapeutic progenitor cell function in the treatment of myocardial ischemia[80], [81]. Paracrine production of SDF-1 by ischemic myocardium has been shown to promote blood vessel formation by implanted c-kit+ CPCs *in vivo* [82]. SDF-1 is thus able to promote angiogenic differentiation in all cell lineages capable of giving rise to endothelium, both in vitro and in vivo.

Previous reports have described the source of SDF-1 as exogenous to the differentiating cell [79], [83]. Our data suggest an autocrine role for SDF-1 in promoting endothelial differentiation of CPCs that is independent of signals from other cells. The mechanism by which intracellular or paracrine SDF-1 interacts with other differentiation signals remains to be determined. SDF-1 may exert proangiogenic effects by inducing VEGF expression [79], [84], activating NO production [78], [85], or initiating a heme oxygenase-dependent signal[74]. Microarray analysis did not reveal significant variations in HIF-1 or VEGF transcripts in undifferentiated CPCs; we also did not find differences in CXCR4 expression, excluding receptor autoregulation or broader upregulation of HIF-1 targets[86], [87], [88]. VEGF is reported to induce SDF-1 expression[66], and HIF-1 is a direct regulator of SDF-1[83], however the lack of clonal variation in these factors means that other epigenetic mechanisms are likely to be responsible for the observed differences in SDF-1 expression among individual progenitor cells.

Although the source of variability remains to be determined, we show here that high endogenous production of SDF-1 promotes vasculogenic differention and vascularization in vivo by CPCs, via a previously unreported autocrine mechanism. SDF-1 may be a useful biomarker for CPCs with enhanced potential for tissue revascularization and a tool for improving vasculogenesis in regenerative cell therapy.

## Materials and Methods

### Materials

Antibodies directed against the following antigens were used to characterize CPCs: Sca-1 (eBioscience); c-Kit, GATA4, troponin I, desmin and vWF (Santa Cruz Biotechnology); smooth muscle actin (Sigma); Flk1 (Cell Signaling); sm22-α and Oct4, (Abcam); Alexa Fluor 488- or 568-conjugated secondary antibodies (Invitrogen). Lipofectamine 2000 (Invitrogen) was used for transfection of 293T cells with viral packaging constructs pCMV-VSV-G and pCMV delta R8.2 (AddGene) and lentiviral pGIPZ vectors encoding anti-SDF-1 shRNA or scrambled non-silencing shRNA (Open Biosystems). For RNA analyses we used the Stem Cell RT2 Profile PCR Array (SABiosciences, Frederick, MD) or standard TaqMan assays (Applied Biosystems). SDF-1 was purchased from R&D Systems. C57/BLKS mice were obtained from Jackson Laboratories. Standard, growth factor-reduced, and high concentration Matrigel basement membrane matrices were obtained from BD Biosciences. Except as noted, all other reagents were obtained from Sigma and were of the highest quality obtainable.

### CPC isolation

All animal procedures were performed according to protocols approved by the University of Miami Institutional Animal Care and Use Committee (#08-202, 08-060, 07-194). 5–6 month old C57 Bl/6 mice were sacrificed, hearts were removed and immediately placed in isolation media (IM) consisting of minimum essential medium (MEM) and penicillin/streptomycin. After careful dissection of the left ventricles, the chambers were gently flushed to remove red blood cells and then cut in 4 parts. Pieces were finely minced in 2 ml of fresh IM, and transferred to a 50 ml centrifuge tube containing 5 ml of pre-warmed 567 U/ml collagenase II (Worthington). Tissues were digested for 30 minutes at 37°C with shaking. The digestion was stopped by addition of 25 ml ice-cold IM and the suspension was then triturated 10x. Undigested heart pieces were allowed to settle, and the supernatant containing CPCs was separated and filtered through a 70 µm mesh strainer.

### CPC growth and cloning

After estimation of cell number, 1×10^7^ cells/ml were incubated with biotin-conjugated anti-Sca-1 antibody for 20 minutes at 4°C in separation buffer (PBS, 0.1% BSA, 2 M EDTA, pH 7.4). Cells were washed 2X and incubated with streptavidin-coated magnetic beads (Dynabeads, Invitrogen Life Science) for 20 minutes at 4°C in separation buffer prior to magnetic sorting according to the manufacturer's instructions. Freshly isolated CPCs were plated in 60 mm bacterial Petri dishes to allow the formation of cardiospheres in CPC medium consisting of F12 medium supplemented with 10% fetal bovine serum (FBS), 10 ng/ml bFGF, 20 ng/ml EGF, 10 ng/ml LIF, 0.5X ITS supplement and antibiotics. After 24–48 h the suspension was distributed into 24 well culture plates and cardiospheres were allowed to attach. All wells were checked daily for proliferating cells. Proliferating CPCs were expanded by gradual transfer from smaller to larger culture dishes. CPCs were initially cloned using cloning rings and then subcloned 1–2 times by serial dilution in 96 well plates. Cell volumes were determined using a Coulter counter (Beckman Coulter).

### CPC differentiation

CPC clones were induced to differentiate by plating cells on gelatin-coated culture dishes in the presence of IMDM medium supplemented with 10% FBS. Culture media was replaced every 48-72 h. Differentiation was followed weekly for a period of 4 weeks. The expression of differentiation markers was determined by real-time PCR analysis on an ABI7900HT Fast sequence detection system using TaqMan primers (Applied Biosystems).

### Matrigel assay

CPC clones were grown in endothelial growth medium (EBM supplemented with serum and growth factors, Lonza) for 1 week prior to plating in 24 well plates coated with Matrigel in endothelial basal medium (EBM supplemented with 0.1% bovine serum albumin) for 5–20 h. Tube formation potential was estimated by measuring the number of tubes per field and tube length using ImageJ software. Each clone was tested in 1–3 independent experiments and at least 5 fields were counted per sample. For some experiments, growth factor-reduced Matrigel was used and cells were supplemented with 175 ng/ml SDF-1, 50 ng/ml AMD3100 and/or vehicle.

### Immunostaining and SDF-1 ELISA

For immunofluorescence, CSCs were washed and fixed in 4% paraformaldehyde for 15 minutes. For intracellular markers, cells were permeabilized with 0.2% Triton X-100 for 5 min. Images were obtained using a Zeiss HBO 100 Axiovert inverted phase/fluorescence microscope. SDF-1 protein expression levels were determined by enzyme-linked immunosorbent assay (ELISA) in 96 well plates coated overnight with 20 µg/ml of whole cell lysates collected from individual clones at different passages. SDF-1 recombinant protein was used as a standard.

### In vivo angiogenesis assay

CPCs in monolayer culture were trypsinized and resuspended in 1 mL of stem cell media to a final concentration of ∼2×10^7^ cells/ml. 500 µL of cell suspension were mixed with 500 µl of growth factor-reduced Matrigel. Control Matrigel plugs were generated by mixing with an equal volume of stem cell media only. Following anaesthesia with ketamine and xylazine, mice received 2 subcutaneous injections of 750 µl of control or CPC-containing Matrigel plugs on each side of the posterior dorsum, following the manufacturer's recommendations. Plugs were harvested and examined at 7 days. Prior to harvest, continuity between host and graft vasculature was determined by intracardiac injection of 1,1′-dioctadecyl-3,3,3′,3′-tetramethylindocarbocyanine perchlorate (DiI) using a previously described method[89]. In brief, mice were sedated, anesthetized by brief exposure to isoflurane, and euthanized by cervical dislocation. A sternotomy was performed and a 25 g needle was inserted into the left ventricular apex, and the right atrium was punctured with an 18 g needle. The vascular system was flushed through the left ventricle using 2 mL of phosphate-buffered saline. 5 mL DiI solution was then injected over 5 minutes, followed by 5 mL 4% paraformaldehyde. Plugs were removed by sharp dissection, then mounted on slides for image acquisition using either a Zeiss HBO 100 Axiovert inverted phase/fluorescence or a LSM510 Axiovert 200 M confocal microscope. Images were exported to.TIFF files, and Adobe Photoshop layers was used for colocalization visualization.

### Lentiviral packaging and transduction for SDF-1 Knockdown

293 T cells (7×10^6^) were plated in 10 cm dishes and cultured overnight in DMEM medium + 10% FBS. The next day, cells were transfected using with 6 µg pGIPZ, 4 µg pCMV deltaR8.2 and 2 µg pCMV-VSV-G per plate. After overnight incubation, the culture media was replaced with fresh DMEM media + 20% FBS. Transfection efficiency was determined by counting GFP-positive cells. Lentivirus particles were collected from supernatants daily beginning 24 h after transfection for 2–3 days and concentrated prior to use. For transduction, CPC clones were plated in a 6 well plate and lentivirus stock added. 4–6 h post-transduction, an additional 2 ml of culture media was added and the cells incubated overnight. At 24–48 h post-transduction cells were examined for GFP expression. Puromycin was used to select clones carrying lentivirus particles and efficiency of SDF-1 knockdown evaluated by ELISA.

### Quantitative realtime PCR

Total RNA was prepared from cells using RNAeasy (Qiagen) or Trizol (Molecular Research Center, Inc) for differentiation or stem cell PCR-arrays, respectively. RNA was quantified by UV spectrophotometry (Nanodrop 1000, Thermo Scientific) and reverse transcribed using an RT2 PCR Array First Strand Kit (SA Bioscience or Applied Biosystems) with random hexamers. cDNA samples were analyzed in duplicate using SYBR green or TaqMan assays on an ABI Prism 7900 HT Fast Sequence Detector System (Applied Biosystems). Ct values were normalized to endogenous Gapdh and Actb. Normalized cts were converted to absolute transcript levels and displayed in heatmap format using Matlab 7.0.4 software (The MathWorks).

### Global Transcription Analysis

For microarray profiling, RNA samples from 5 clones were isolated using Trizol (Molecular Research Center, Inc.), purified by passage through Qiagen RNeasy columns according to the manufacturer's instructions, and labeled for hybridization to Affymetrix Mouse Genome 430 2.0 Arrays using standard protocols. Briefly, arrays were pre-hybridized for 10 minutes at 45°C, after which labeled samples were added and hybridized for 16 hours at 45°C. The arrays were stained and washed according to Affymetrix Fluidics Station 450 protocol (EukGEWS2v5_450). Hybridization was documented using a GeneChip Scanner 3000 7G and validated with Affymetrix Microarray Suite version 5.0 (MAS 5.0) software. Pearson correlation coefficients demonstrated high reproducibility. All data is MIAME compliant and has been submitted to the National Center for Biotechnology Information GEO database, accession #GSE24828.

### Statistics

Microarray statistical analysis was performed using GeneSpring 7.2 software (Silicon Genetics, Redwood City, CA). Normalized expression values were calculated by the GCRobust Multi-array Average (GC-RMA) method. The Gene Ontology annotation tool was used to generate functional classifications, and hierarchical clustering was performed using Pearson correlation as a similarity measure and average linkage as a clustering algorithm. Other statistical analyses, including linear regression and one-way ANOVA with Newman-Keuls and Bonferroni multiple comparisons tests were performed using GraphPad Prism 4.0c for Macintosh (GraphPad Software, San Diego CA USA, www.graphpad.com).

## Supporting Information

Figure S1 **Growth curves of 6 different cardiac progenitor cell clones.** The growth pattern of CPC clones was followed for a period of 6 days and doubling time was estimated to occur every 24–48 hours. Growth patterns were similar among most clones tested, with one exception. Values correspond to the number of viable cells as determined by trypan blue exclusion.
(PDF)

Figure S2 **Weak correlation between FLK-1 expression and vasculogenic potential.** (A) FLK1 expression was measured by Western blot in 12 undifferentiated CPC clones as shown and in HUVECs (ECs). (B) Quantitation of Flk 1 expression for each clone, normalized to GAPDH. (C) Correlation (R) between FLK-1 expression and vasculogenic index (see Table S1).
(PDF)

Figure S3 **SDF-1 Knockdown in Cardiac progenitor cells.** SDF-1 was knocked down by lentiviral transduction of CPCs using vectors expressing SDF-1 shRNA. Subclones expressing one of three different shRNAs (E1, H7, D8) or a scrambled control shRNA (NS) were generated from the same parental clone. Original viral transduction dose (particles/cell) shown on abscissa.
(PDF)

Figure S4 **Angiogenesis in Matrigel dermal inserts of CPCs.** Low growth factor Matrigel plugs were implanted without cells (A), with weakly vasculogenic CPC clone 30C1 (B, D), and with vasculogenic clone 11B (E–H). A–C and G show overlay of brightfield and epifluorescent images. Original magnification A, B, E, F = 10x, C, D, G, H = 32X.
(PDF)

Table S1 **GO functions that cluster CPC clones with similar morphology.**
(PDF)
