# Supplementary material for: Heterogeneity in SDF-1 Expression Defines the Vasculogenic Potential of Adult Cardiac Progenitor Cells
Source: PLoS One. 2011 Aug 24;6(8):e24013. doi: 10.1371/journal.pone.0024013 (PMC3161114; doi:10.1371/journal.pone.0024013)
Supplement: Figure S1 — Growth curves of 6 different cardiac progenitor cell clones. The growth pattern of CPC clones was followed for a period of 6 days and doubling time was estimated to occur every 24–48 hours. Growth patterns were similar among most clones tested, with one exception. Values correspond to the number of viable cells as determined by trypan blue exclusion. (PDF) [file pone.0024013.s001.pdf]

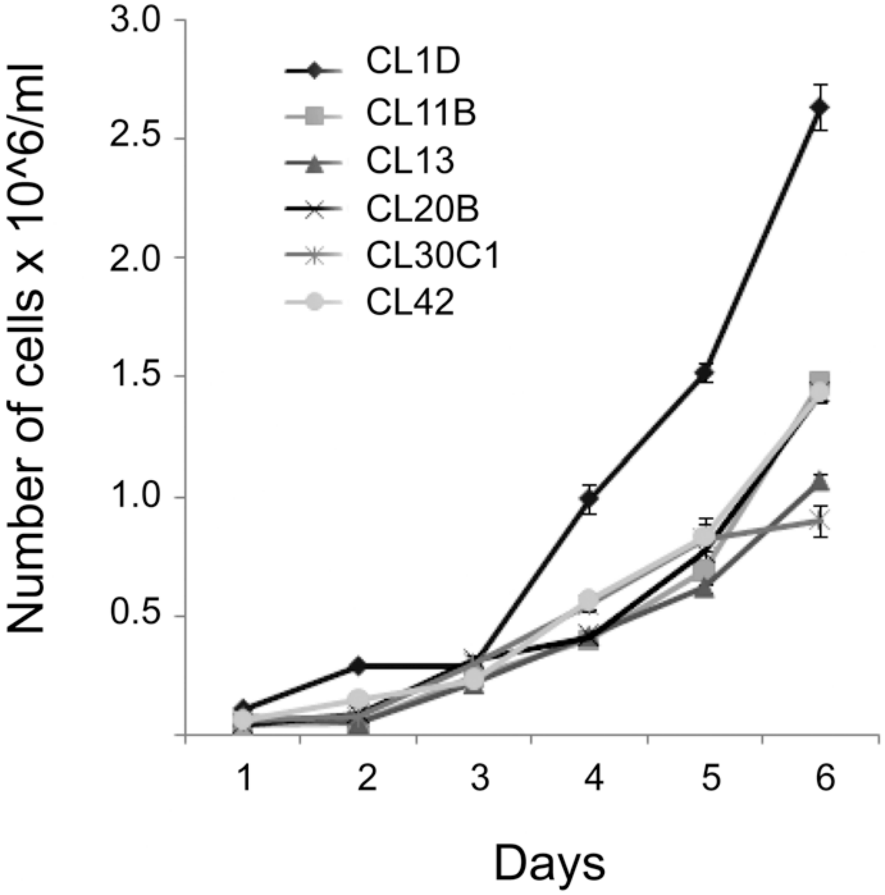

Figure S1. Growth curves of 6 different cardiac progenitor cell clones. The growth pattern of CPC clones was followed for a period of 6 days and doubling time was estimated to occur every 24-48 hours. Growth patterns were similar among most clones tested, with one exception. Values correspond to the number of viable cells as determined by trypan blue exclusion.
