# Supplementary material for: Heterogeneity in SDF-1 Expression Defines the Vasculogenic Potential of Adult Cardiac Progenitor Cells
Source: PLoS One. 2011 Aug 24;6(8):e24013. doi: 10.1371/journal.pone.0024013 (PMC3161114; doi:10.1371/journal.pone.0024013)
Supplement: Figure S2 — Weak correlation between FLK-1 expression and vasculogenic potential. (A) FLK1 expression was measured by Western blot in 12 undifferentiated CPC clones as shown and in HUVECs (ECs). (B) Quantitation of Flk 1 expression for each clone, normalized to GAPDH. (C) Correlation (R) between FLK-1 expression and vasculogenic index (see Table S1). (PDF) [file pone.0024013.s002.pdf]

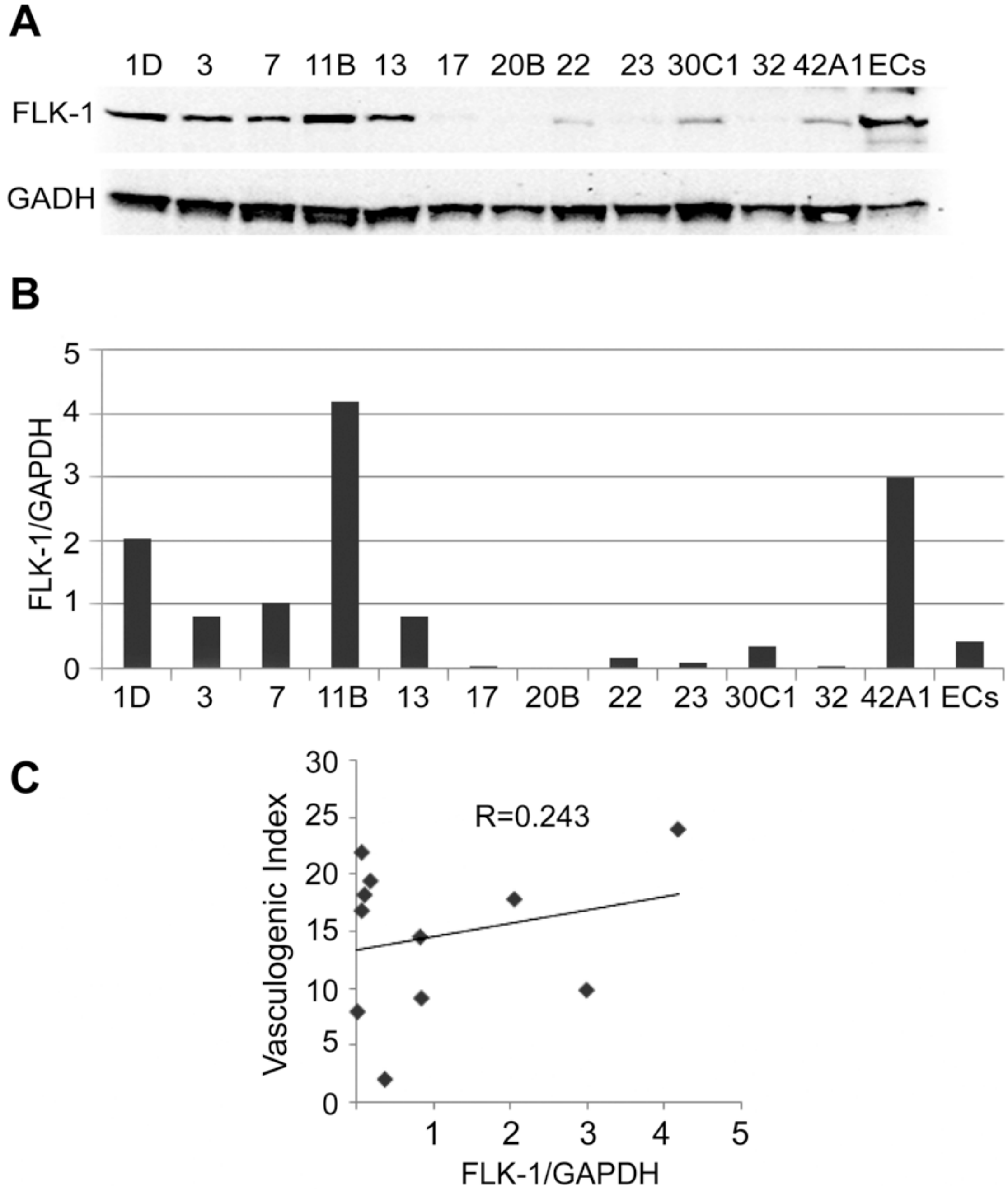

Figure S 2. Weak correlation between FLK-1 expression and vasculogenic potential. (A) FLK1 expression was measured by Western blot in 12 undifferentiated CPC clones as shown and in HUVECs (ECs). **(B)** Quantitation of Flk 1 expression for each clone, normalized to GAPDH. **(C)** Correlation (R) between FLK-1 expression and vasculogenic index (see Supplemental Table 1).
