# Supplementary material for: Heterogeneity in SDF-1 Expression Defines the Vasculogenic Potential of Adult Cardiac Progenitor Cells
Source: PLoS One. 2011 Aug 24;6(8):e24013. doi: 10.1371/journal.pone.0024013 (PMC3161114; doi:10.1371/journal.pone.0024013)
Supplement: Figure S3 — SDF-1 Knockdown in Cardiac progenitor cells. SDF-1 was knocked down by lentiviral transduction of CPCs using vectors expressing SDF-1 shRNA. Subclones expressing one of three different shRNAs (E1, H7, D8) or a scrambled control shRNA (NS) were generated from the same parental clone. Original viral transduction dose (particles/cell) shown on abscissa. (PDF) [file pone.0024013.s003.pdf]

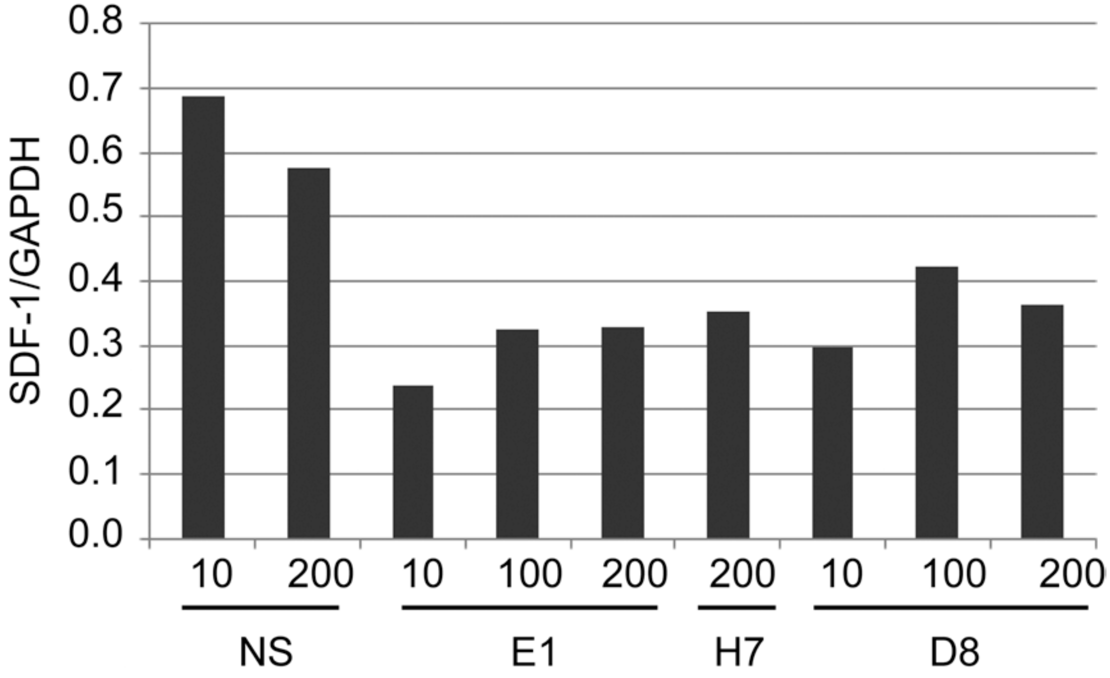


**Figure S3. SDF-1 Knockdown in Cardiac progenitor cells.** SDF-1 was knocked down by lentiviral transduction of CPCs using vectors expressing SDF-1 shRNA. Subclones expressing one of three different shRNAs (E1, H7, D8) or a scrambled control shRNA (NS) were generated from the same parental clone. Original viral transduction dose (particles/cell) shown on abscissa.
