# Supplementary material for: Heterogeneity in SDF-1 Expression Defines the Vasculogenic Potential of Adult Cardiac Progenitor Cells
Source: PLoS One. 2011 Aug 24;6(8):e24013. doi: 10.1371/journal.pone.0024013 (PMC3161114; doi:10.1371/journal.pone.0024013)
Supplement: Figure S4 — Angiogenesis in Matrigel dermal inserts of CPCs. Low growth factor Matrigel plugs were implanted without cells (A), with weakly vasculogenic CPC clone 30C1 (B, D), and with vasculogenic clone 11B (E–H). A–C and G show overlay of brightfield and epifluorescent images. Original magnification A, B, E, F = 10x, C, D, G, H = 32X. (PDF) [file pone.0024013.s004.pdf]

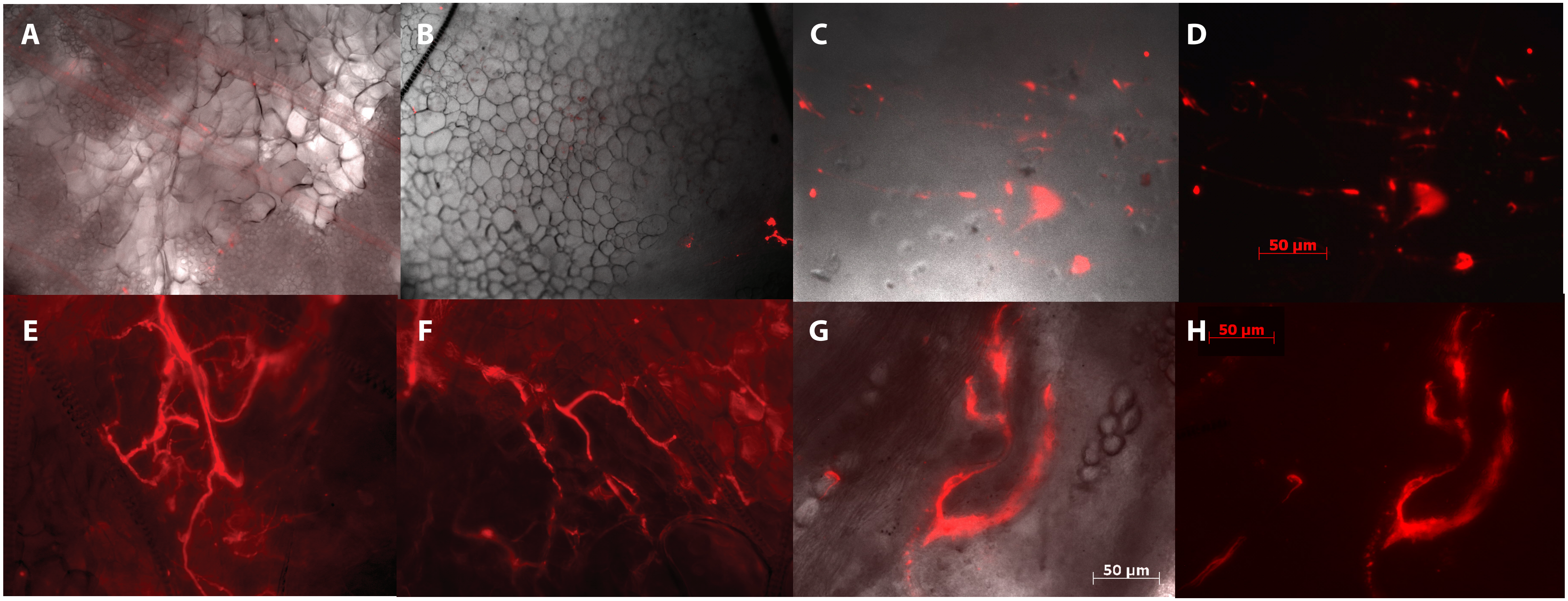

Figure S4. Angiogenesis in Matrigel dermal inserts of CPCs. Low growth factor Matrigel plugs were implanted without cells (A), with weakly vasculogenic CPC clone 30C1 (B-D), and with vasculogenic clone 11B (E-H). A-C and G show overlay of brightfield and epifluorescent images. Original magnification A, B, E, F = 10x, C, D, G, H = 32X.
