# Supplementary material for: Heterogeneity in SDF-1 Expression Defines the Vasculogenic Potential of Adult Cardiac Progenitor Cells
Source: PLoS One. 2011 Aug 24;6(8):e24013. doi: 10.1371/journal.pone.0024013 (PMC3161114; doi:10.1371/journal.pone.0024013)
Supplement: Table S1 — GO functions that cluster CPC clones with similar morphology. (PDF) [file pone.0024013.s005.pdf]

Table S1. GO functions that cluster CPC clones with similar morphology.

GO Function	Gene Name
**Antibody dependent cellular toxicity**	Fcgr3
	Lgh-4
	Lgh-1a
**Cell death**	Aplp1
	Clu
	Tnfrsf1a
**Germ cell migration**	CXCl12
	CXCR4
**Metal ion homeostasis**	Mt3
	Mt1
**Microtubule stabilization**	Clasp1
	Clasp2
	Hdac6
	Mapt
	Rnf30
**Morphogenesis of an epithelial sheet**	Dag1
	Lama1
	Notch2
**Neuronal migration**	Pxmp3
	Pex5
	Pex5

**Nitric oxide mediated signal transduction**	Ddah2
	Mt1
**Nucleolus organization and biogenesis**	Nolc1
	Pes1
**Peroxisome organization and biogenesis**	Abcd3
	Abcd4
	Peci
	Pex3
	Pex5
	Pex7
	Pex11a
	Pex11b
	Pex11a
	Pex16
	Pex19
	Pipox
	Pxmp2
	Pxmp3
**Regulation of cell adhesion**	Cmar
	Prlr
	Pscd2
	Pscdbp
	Stat5a
	Stat5b
**Response to heat**	A030005K1

	Dnajb3
	Hspa1b
	Hspa1l
	Hspa2
	Hspa8
	Hspa9a
	Hspb1
	Hsp105
	Krtap5-1
	Stk4
	4833428E2
**Retinoid metabolism**	Adh5
	Adh1
	Rdh1
	Rbp2
	Rbp1
**Sulfate assimilation**	Papss1
	Sult2b1
	Sult1d1
